# Neural networks for increased accuracy of allergenic pollen monitoring

**DOI:** 10.1038/s41598-021-90433-x

**Published:** 2021-05-31

**Authors:** Marcel Polling, Chen Li, Lu Cao, Fons Verbeek, Letty A. de Weger, Jordina Belmonte, Concepción De Linares, Joost Willemse, Hugo de Boer, Barbara Gravendeel

**Affiliations:** 1 Naturalis Biodiversity Center, Leiden, The Netherlands; 2 Leiden Institute of Advanced Computer Science (LIACS), Leiden, The Netherlands; 3 Department of Pulmonology, Leiden University Medical Center, Leiden, The Netherlands; 4 Institute of Environmental Sciences and Technology (ICTA-UAB), The Universitat Autònoma de Barcelona, Bellaterra, Cerdanyola del Vallès, Spain; 5 Microbial Sciences, Institute of Biology, Leiden, The Netherlands; 6 Natural History Museum, University of Oslo, Oslo, Norway

**Keywords:** Atmospheric science, Transmission light microscopy, Asthma, Computer science, Plant sciences, Environmental sciences

## Abstract

Monitoring of airborne pollen concentrations provides an important source of information for the globally increasing number of hay fever patients. Airborne pollen is traditionally counted under the microscope, but with the latest developments in image recognition methods, automating this process has become feasible. A challenge that persists, however, is that many pollen grains cannot be distinguished beyond the genus or family level using a microscope. Here, we assess the use of Convolutional Neural Networks (CNNs) to increase taxonomic accuracy for airborne pollen. As a case study we use the nettle family (Urticaceae), which contains two main genera (*Urtica* and *Parietaria*) common in European landscapes which pollen cannot be separated by trained specialists. While pollen from *Urtica* species has very low allergenic relevance, pollen from several species of *Parietaria* is severely allergenic. We collect pollen from both fresh as well as from herbarium specimens and use these without the often used acetolysis step to train the CNN model. The models show that unacetolyzed Urticaceae pollen grains can be distinguished with > 98% accuracy. We then apply our model on before unseen Urticaceae pollen collected from aerobiological samples and show that the genera can be confidently distinguished, despite the more challenging input images that are often overlain by debris. Our method can also be applied to other pollen families in the future and will thus help to make allergenic pollen monitoring more specific.

## Introduction

Pollen allergies are on the rise globally, with worldwide approximately 10–30% of adults and 40% of children affected^1,2^. For patients the symptoms include a runny nose, sneezing and itchy eyes, mouth or skin. Control measures and medication are readily available, but to alleviate the symptoms most efficiently, exposure to allergens should be kept to a minimum^3^. Therefore, for more and more people, fast and accurate monitoring of airborne pollen provides an essential early warning system^4,5^. Pollen concentrations in the air are monitored using samplers that collect airborne pollen on sticky tape, e.g. Hirst type samplers^6^. These tapes are microscopically inspected for their pollen content, a process that requires highly trained specialists. Moreover, although the allergenic pollen from some plants can be monitored at the species level (e.g. species of plantain, *Plantago* L.^7^), many other pollen grains cannot be accurately identified to this level. In many taxa, only a genus- or family-level identification is possible using current microscopic methods^8^. This is problematic since different species and even genera within the same family can possess very different allergenic profiles. An extra challenging factor in airborne pollen identification from Hirst samples is that they are collected directly from the air. In contrast to pollen grains that have been acetolyzed ^9^, these pollen grains still contain all organic material, and defining features are less apparent^10^.

This identification challenge is exemplified in the case of the nettle family (Urticaceae). Pollen grains produced by all species from the genus *Urtica* L. (stinging nettles) have a low allergenic profile^11^, while pollen from several species of *Parietaria* L. (pellitory) is a major cause of hay fever and asthma, in particular *P. judaica* L. and *P. officinalis* L.^12,13^. These pellitory species are native to the Mediterranean, but throughout the second half of the twentieth century, a range expansion occurred through north-eastern Europe, the Americas and Australia as a result of anthropogenic distribution and climate change^14,15^. *Parietaria* sensitization is highly different per geographic area, but has been reported to reach 80% in southern Italy while a value of 13% was found in the United Kingdom^16^. Species of *Parietaria* flower throughout the year but their main flowering peaks occur in May–June and August-October, which overlaps with the flowering season of *Urtica* species (June–October)^17^. Cross-reactivity is present between species of *Parietaria*, but is absent between the genera *Urtica* and *Parietaria*^11,18,19^. *Parietaria* pollen is microscopically indistinguishable from that of *Urtica* and their contribution to the total airborne pollen load is currently not assessed in either native or expanded range^20^.

Pollen grains from *Urtica* and *Parietaria* species have a simple morphology: they are small (~ 11–20 µm), rounded to slightly ellipsoidal tri-, tetra- or zonoporate with a psilate to scabrate surface ornament and small pores. Most species have an annulus around the pore, i.e. a thickening of the otherwise very thin exine and a germination area called the oncus (lens-shaped body located in the apertural region)^7^. The only species of Urticaceae that can be distinguished in aerobiological samples is *Urtica membranacea* due to its small size (~ 10–12 μm) and a high number of pores (usually more than six^21^. The main difference between the pollen of *Urtica* and *Parietaria* are the slightly smaller size and coarser surface ornamentation of *Parietaria*, and a more angular outline and more pronounced annulus of *Urtica*^22^.

Despite recent advances in innovative technologies, palynology is still largely an image-based discipline^23^. Therefore, automating this process currently receives a lot of attention. Automatic classification using manually selected pollen-specific features has typically resulted in relatively low classification success (see e.g.^24,25^). However, recent studies applying advances using deep learning have been very promising^26–29^. Neural networks have been used successfully to manage both the tasks of differentiating pollen from non-pollen debris as well as correctly identifying different taxa (for an overview please refer to ^23^). Automatic image recognition can, however, also be used to improve identification of pollen taxa that are difficult to distinguish using traditional methods. Subtle variations in morphology that are not readily apparent through microscopic investigation may be consistently detected by neural networks. This has for example been shown for the highly similar pollen of black spruce (*Picea mariana* (Mill.) Britton, Sterns & Poggenb.) and white spruce (*Picea glauca* (Moench) Voss) using machine learning ^30^ and for pollen of ten species of the thistle genus *Onopordum* L. using an artificial neural network^31^. Recent advances have also been made in the field of aerobiological samples with for example the distinction of anomalous from normal pollen grains of common hazel (*Corylus avellana* L.) ^32^. However, neural networks have so far not been tested for improvement of taxonomic resolution in unacetolyzed pollen in aerobiological samples.

Here we use Convolutional Neural Networks (CNNs) to distinguish morphologically similar, unacetolyzed pollen from the nettle family. We collect pollen from all species of Urticaceae present in the Netherlands (*Urtica dioica, U. membranacea, U. urens, Parietaria judaica* and *P. officinalis*). The pollen was collected from several sources for each species, freshly collected as well as from herbaria, and used to create a pollen image reference dataset. We compare the results of CNNs trained from scratch with those from pre-trained CNNs using transfer learning. Because of the limited size of the pollen image dataset, pre-training the CNN on a publicly available image database can help to recognize the distinguishing features of pollen grains such as pores, texture and shape.

We test both the deep CNN VGG16 and the faster CNNs MobileNetV1 and V2, and optimize the performance using data augmentation. The model is then applied to unknown Urticaceae pollen from three aerobiological samples with high Urticaceae pollen counts. We use one sample from the Leiden University Medical Centre (LUMC), Leiden, the Netherlands as well as one sample each from Lleida and Vielha, Catalonia, Spain (ICTA-UAB). In the Netherlands, stinging nettles (*Urtica*) are highly abundant and therefore it is expected that most Urticaceae pollen will be from this genus. *Urtica* is also expected to be dominant in Vielha, while in the direct surroundings of Lleida, *Parietaria* is very abundant.

The main objectives of this study are (1) to see whether a CNN model can distinguish morphologically similar unacetolyzed pollen of two common genera and a species in the Urticaceae family that have highly differing allergenic profiles; (2) to test whether the trained model can be successfully applied on aerobiological samples containing more complex and for the model before unseen input images.

## Results

### Model performance

In this study three different CNNs were tested on unacetolyzed pollen of Urticaceae which cannot currently be separated by specialists. The highest accuracy of the models using the three classes *Urtica, Parietaria* and the species *Urtica membranacea* was obtained using fivefold cross-validation (i.e. 80% training, 20% validation) with either VGG16 (98.61%) or MobileNetV2 (98.76%) (Table 1). Since VGG16 and MobileNetV2 had very similar performance, we trained these two models two more times to see which model performed more consistently. The mean accuracy after three repetitions was 98.50% for VGG16 with 0.145% standard deviation and 98.45% for MobileNetV2 with relatively higher standard deviation (0.289%). The models trained from scratch showed significant lower accuracy for MobileNetV1 and V2 (both < 89%) while this value was 96.29% for VGG16.

**Table 1:** Performance comparisons of VGG16, MobileNetV1 and MobileNetV2, comparing models trained from scratch with pre-trained models as well as fivefold versus tenfold cross-validation.0 .

CNN	Method	Cross-validation	Accuracy (%)	Precision	Recall	F1-score
VGG16	From scratch	Fivefold	96.29	0.9632	0.9629	0.9629
		Tenfold	96.14	0.9616	0.9614	0.9614
	Pre-trained	Fivefold	**98.61**	0.9861	0.9861	0.9861
		Tenfold	**98.30**	0.9831	0.9830	0.9830
MobileNetV1	From scratch	Fivefold	84.54	0.8454	0.8454	0.8454
		Tenfold	86.40	0.8640	0.8640	0.8641
	Pre-trained	Fivefold	**98.15**	0.9815	0.9815	0.9816
		Tenfold	**98.15**	0.9815	0.9815	0.9815
MobileNetV2	From scratch	Fivefold	87.64	0.8769	0.8764	0.8763
		Tenfold	88.56	0.8857	0.8856	0.8856
	Pre-trained	Fivefold	**98.76**	0.9877	0.9876	0.9876
		Tenfold	**98.45**	0.9849	0.9845	0.9846

Values in bold represent the highest accuracy scores obtained for each of the three models.

**Figure 1:**
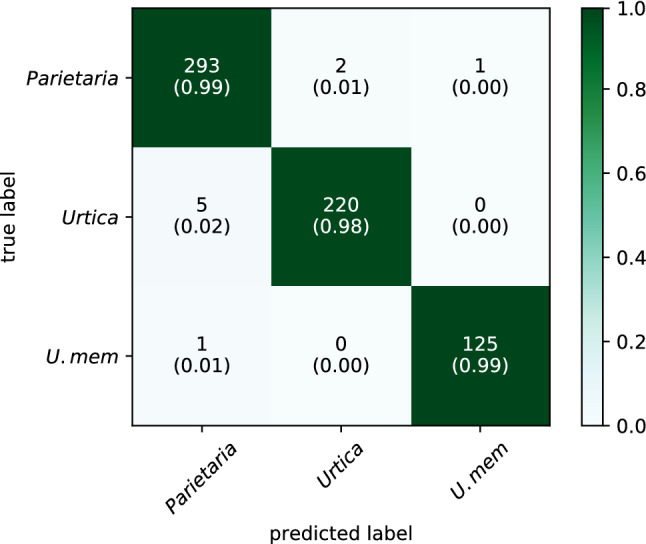
Confusion matrix of results of pre-trained VGG16 using 80% of the images for training and 20% for testing. Numbers represent the actual number of correctly recognized images while those between brackets represent the ratio of correctly classified images. *U.mem* = *Urtica membranacea*.

As the CNNs showed equally high accuracies with the pre-trained method (> 98%), we applied the more consistent VGG16 model using fivefold cross-validation and show the results here. The model accurately identified pollen to the genus level for 97.8% of the test images for *Urtica* and 99.0% for *Parietaria* (Fig. 1). For *Parietaria* three images were misclassified, while five were misclassified for *Urtica* (all to *Parietaria*). The species *Urtica membranacea* was confidently distinguished from all other Urticaceae species (99.2%), but distinction at the species-level was not possible for any of the other *Urtica* and *Parietaria* species. This is because the distinguishing features of pollen from these species (e.g. exine ornamentation) could not be resolved in the used image projections.

**Figure 2:**
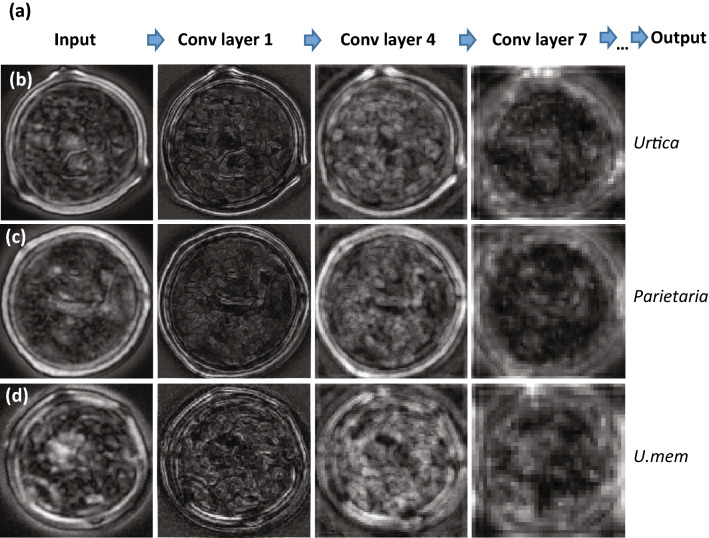
Feature maps. (a) simplified view of the VGG16 model showing three convolutional layers. (**b**–**d**) Feature maps of Urticaceae pollen grains from the standard deviation projection created using ImageJ, that were confidently distinguished by the CNNs. (**b**) *Urtica urens*, (**c**) *Parietaria judaica* and (**d**) *Urtica membranacea*. Activation levels are indicated with white indicating high activation and black very low/no activation.

For all species, pollen grains were collected from a minimum of four different plants. Looking at the raw pollen images from the different plants, we identified intra-specific differences that result from natural variability within each species. To test whether the CNNs learned the pollen-specific distinguishing features rather than sample-specific details, we produced feature maps for the VGG16 model (Fig. 2b–d). Despite the highly variable input images of unacetolyzed pollen from different plants, the model consistently learned features such as edges in the first convolutional layers, while finer features such as pores and annuli were learned in deeper layers.

### Application to test cases

Table 2 shows the results of the CNN on unknown and before unseen Urticaceae pollen from an aerobiological sample from Leiden, the Netherlands, as well as from Lleida and Vielha, Catalonia, Spain. We set the identification threshold at a value of 60% as derived from the model test images, and therefore the CNN also returned unknown images (see Supplementary Table S1 for the full results). For the sample from Leiden, 85.7% of the Urticaceae pollen was identified as *Urtica*, with only a minor presence of *Parietaria* (4.5%). The sample from Lleida shows dominance of *Parietaria* pollen grains (81.0%) while 14.3% of the Urticaceae pollen grains were classified as *Urtica*. Finally, for Vielha we find a mixture of ~ 70% *Urtica* and ~ 20% *Parietaria.* No *Urtica membranacea* pollen grains were identified in any of the samples. On average, unknown images account for 8.7% of the total images when using 60% identity threshold. When using a stricter identity threshold (e.g. 70%, see Table 2), the unknown image category increases to an average value of 13.5%.

**Table 2:** Results of the deep learning model VGG16 on Urticaceae pollen from an area representing 10% of the total deposition area of Hirst-type aerobiological samples from Leiden (the Netherlands), Lleida and Vielha (both Catalonia, Spain).

Sample location	Date collected	No. Pollen	% *Urtica*	% *Parietaria*	% *U. mem*	% Unknown	Identity threshold
Leiden, NL	23/08/2019	112	**85.7**	4.5	0	9.8	60%
Lleida, SP	16/06/2019	63	14.3	**81.0**	0	4.8	60%
Vielha, SP	09/08/2019	26	**69.2**	19.2	0	11.5	60%
Leiden, NL	23/08/2019	112	**83.0**	3.6	0	13.4	70%
Lleida, SP	16/06/2019	63	12.7	**79.4**	0	7.9	70%
Vielha, SP	09/08/2019	26	**69.2**	11.5	0	19.2	70%

Values in bold represent the highest accuracy scores obtained for each of the three classes.

The threshold for identification was tested at 60% and 70%. Images that were classified below this level were classified as unknown. *U.mem* = *Urtica membranacea*.

## Discussion

This study demonstrates incorporating neural networks to increase the taxonomic resolution of pollen grain identifications in aerobiological samples. The feature maps in Fig. 2 show that the trained deep learning model VGG16 looks at the traditionally used morphological features to distinguish *Urtica* from *Parietaria* pollen grains. The characteristic thickening of the exine around the pores of *Urtica* shows the highest activation in the deeper convolutional layers. The distinct thickening is missing in *Parietaria* pollen, and the model instead focuses on the pollen outline. As expected, the only species to be distinguished by our model is *Urtica membranacea* which shows a slightly angular outline due to the larger numbers of pores (Fig. 2d). For the other species used in this study, no distinction was possible even though it has been shown that pollen from species of *Urtica* (*U. dioica* and *U.* *urens*) (Fig. 2b) and *Parietaria* (*P. judaica* and *P. officinalis*) (Fig. 2c) can be separated based on differences in their exine ornamentation^22^. These differences can, however, only be imaged using specialized microscopy methods such as SEM or phase-contrast imaging, and are very hard to visualize using brightfield microscopy. Furthermore, these features are obscured when pollen grains are not acetolyzed. For our purposes, this species level distinction is not relevant as no known differences in allergenicity are known between either the species of *Urtica*^11^ or *Parietaria*^18^.

Similar to a recent study comparing pollen image classification methods, we found that using a pre-trained CNN consistently outperforms the models trained from scratch^33^. This transfer learning approach is also used by many other recent studies on deep learning of pollen images, mainly because of the limited amount of training images^26–29,34,35^. Still, we find that the VGG16 model trained from scratch achieves a high accuracy of 96.29%. This is because compared to the MobileNets, VGG16 architecture has more and deeper parameters. The MobileNets have less training parameters making them much lighter and faster, and the high accuracies found here indicate that they can be used as a light-weight alternative. In our models the amount of False Positives (FP) is nearly equal to the amount of False Negatives (FN) which is why recall, precision and F1-score were very similar.

This is the first time deep learning has been used to increase the taxonomic accuracy of unacetolyzed pollen identifications. The models represent a significant improvement of earlier attempts in distinguishing Urticaceae pollen using automatic image classification. In a previous study using hand-designed shape and texture features, pollen from three Urticaceae species could be distinguished from another with an 89% accuracy^36^, though only a small image dataset was used to train the model (i.e. 100 images per species). Similar results were obtained by ^24^ where shape features were used with a minimum distance classifier to obtain a 86% accuracy between three species of Urticaceae. Because not all species of Urticaceae were included and a low amount of training images was used, these studies have limited applicability to the highly diverse pollen encountered in aerobiological slides. Furthermore, for both studies the trained model was tested on real case examples and only *Urtica membranacea* was successfully identified (> 98%). The other two classes (*Urtica*) and (*Parietaria*) showed very high error rates (up to 44.4%)^24^. This could be because the model was not trained with sufficient variability. Because we trained the models with pollen from various sources and used data augmentation, they had a better generalizing capability.

Deep learning models have shown similar accuracy rates to ours on larger and more varied pollen datasets as well, but these either focussed on the family level^37–39^ or on insect-collected pollen for honey analysis^26–28^. Increasing the taxonomic resolution of pollen grains has been achieved by incorporating an extensively trained deep learning model with super-resolution microscopy on a case study of fossil pollen^35^. Similarly, incorporating SEM images has been found to allow for highly accurate distinction of pollen types^29^. These microscopy methods, however, are often much more expensive than using light microscopy and require extensive sample preparation. Moreover, nearly all of these studies work with acetolyzed pollen that allow easier recognition of distinguishing features, and used pollen collected from a single location.

To validate our model, we tested it on Urticaceae pollen from aerobiological samples collected from different locations in Spain and the Netherlands. Most of the pollen grains from the sample from Leiden, the Netherlands were identified by the deep learning model as *Urtica*, with only a low number of images identified as *Parietaria*. While *Parietaria* plants are relatively abundant around the sampling location in Leiden and were flowering on the chosen date, its pollen is most likely simply outnumbered by the much larger number of nettles in the area. For Lleida (Catalonia), where pellitory plants are abundantly present, *Parietaria* pollen grains dominated the assemblage, while the sample from Vielha showed a mixed assemblage. The number of unknown images was the highest for the sample from Vielha (11.5%), which is most likely the result of the presence of more debris on the pollen grains making a certain identification impossible. In all aerobiological slides, debris on top of or below the pollen grains was observed in different focal plains. Nevertheless, the model still successfully classified most of the pollen grains, and in most cases with high confidence (Supplementary Table S1). This shows the potential broad application of this method and opens up opportunities to study both seasonal as well as long-term yearly dynamics of *Parietaria* versus *Urtica* abundance of airborne pollen, as well as using this method to distinguish other morphologically similar species of allergenic importance from different families (e.g. Betulaceae, Amaranthaceae, Oleaceae). To further improve the generalization of this classification system, future work will focus on increasing the amount of training images from variable sources. Furthermore, more elaborate techniques like regularization will be considered to improve the variability in the image dataset ^40^. Since for allergenic pollen monitoring reducing the amount of false negatives (i.e. increasing recall) is particularly important, more models will be tested to identify the best recall values.

A limitation of our method is that currently pollen from aerobiological slides have to be located manually. It has already been shown that automating this process is feasible, e.g. using a deep learning approach^41^. In other systems like the commercially available Classifynder system, pollen are automatically located and imaged using darkfield imaging after which a simple neural network classifies the pollen^42^. This is also the case for the BAA500 system used by, e.g. Oteros et al.^43^, that was particularly developed for recognizing and classifying unacetolyzed airborne pollen for hay fever predictions. Lastly, using a CNN and digital holography on pollen grains directly from the air (i.e. unacetolyzed) showed great promise in quantifying pollen automatically to the family level^44^. While these systems achieve automated and accelerated pollen counting, our method instead particularly increases the accuracy of information useful for allergy prevention by making it more specific.

## Conclusions

In conclusion, using a combination of an image-processing workflow and a sufficiently trained deep learning model, we were able to differentiate unacetolyzed pollen grains from two genera and one species in the nettle family. These are genera that are indistinguishable with current microscopic methods but possess different allergenic profiles, and thus the ability to differentiate them is of medical significance. Our method can be more broadly applied to distinguish pollen from similarly challenging allergenic plant families and can help in producing more accurate pollen spectra to improve the forecasts for allergy sufferers.

## Material and methods

**Figure 3:**
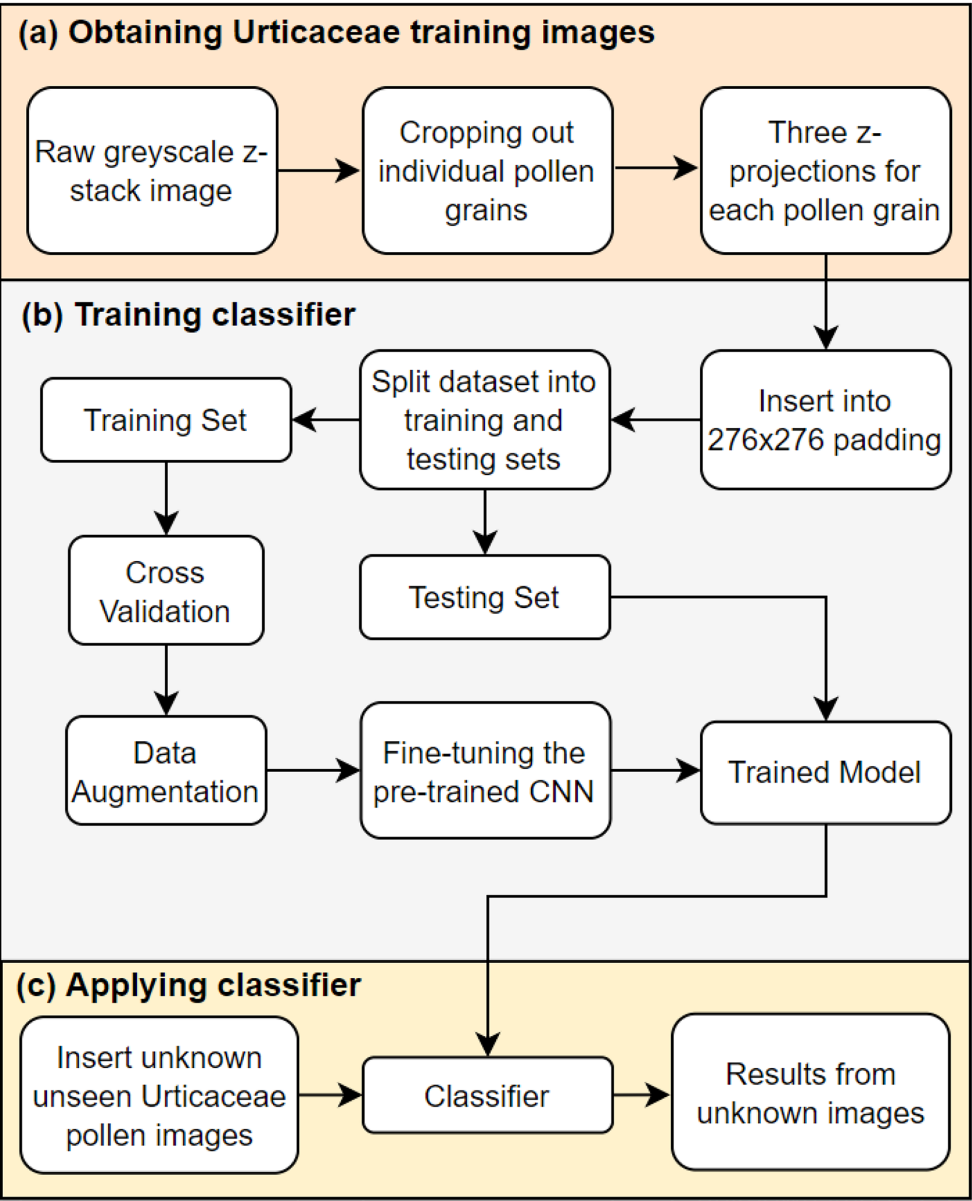
Flowchart showing the pollen image classification process. (**a**) Reference pollen image capture using the custom Fiji macro Pollen_Projector. (**b**) Images were inserted into a fixed frame and split into training and testing sets. The training set was used for cross-validation and data augmentation (flip, brightness) so as to train the CNNs VGG16, MobileNetv1 and MobileNetv2. Results from the models trained from scratch are compared to results from transfer learning on pre-trained models. (**c**) Images from before unseen unknown Urticaceae pollen grains are fed to the resulting classifier. Created using https://app.diagrams.net/.

A flowchart has been constructed to visualize all the steps in the Urticaceae pollen image classification process (Fig. 3). Details on the individual steps are described in this section.

### Collection of pollen

Pollen grains were collected from all five species of Urticaceae found in the Netherlands. In the genus *Urtica*, the native species *U. dioica* L. (common nettle) and *U. urens* L. (small nettle) are ubiquitous in nitrogen rich moist areas, ditches, woodlands, disturbed sites and roadsides. The exotic Mediterranean species *U. membranacea* is rarely encountered, though is included in this study since its range is expected to increase due to the effects of global warming. The genus *Parietaria* is represented in the Netherlands by the species *P. judaica* L. (pellitory of the wall) and *P. officinalis* L. (upright pellitory) that both occupy rocky substrates, mainly in the urban environment^15^. Moreover, *P. judaica* has shown a big increase in abundance over the past decades, e.g. in the Netherlands (Supplementary Fig. S1), but also in many other parts of the world.

Pollen from all Urticaceae species was either freshly obtained or collected from herbarium specimens (Naturalis Biodiversity Center). Fresh material was collected with the help of an experienced botanist (Barbara Gravendeel) in the direct surroundings of Leiden and The Hague during the nettle flowering seasons of 2018 and 2019. All newly collected plant specimens have been vouchered and were deposited in the herbarium of the Naturalis Biodiversity Center (L.3993376–L.3993387) (Supplementary Table S2). Original taxonomic assignments for the herbarium specimens were verified using identification keys and descriptions^45^. A minimum of four different plants were sampled per species, from different geographical locations to cover as much of the phenotypic plasticity in the pollen grains as possible and reflect the diversity found on aerobiological slides.

To produce palynological reference slides, thecae of open flowers were carefully opened on a microscopic slide using tweezers. A stereo microscope was mounted in a fume hood to avoid inhalation of the severely allergenic pollen of *Parietaria* species. Non-pollen material was manually removed to obtain a clean slide. The pollen grains were mounted using a glycerin:water:gelatin (7:6:1) solution with 2% phenol and stained with Safranin (0.002% w/v). These represent the same conditions as used in airborne pollen analysis on pollen collected with a Hirst type sampler. Cover slips were secured with paraffin.

### Pollen image capture

A total of 6472 individual pollen grains were scanned from the five different species of Urticaceae. The number of images for each species varied between 1055 and 1670 (Supplementary Table S2). The images were divided into three classes, namely *Urtica* (*U. dioica* + *U. urens*), *Parietaria* (*P. judaica* + *P. officinalis*) and *U. membranacea*. The system used for imaging was a Zeiss Observer Z1 (inverted microscope) linked to a Hamamatsu EM-CCD Digital Camera (C9100), located at the Institute of Biology Leiden (IBL). Grayscale images were used, since the pollen was stained to increase contrast and not for species recognition.

The imaging procedure was as follows: on each microscope reference slide containing only pollen of one species of Urticaceae, an area rich in pollen was identified by eye and this area was automatically scanned using multidimensional acquisition with the Zeiss software Zen BLUE. For areas that were very rich in pollen, a user-defined mosaic was created consisting of all the tiles to be scanned (e.g. 20 × 20 tiles), while a list of XY positions was used for microscopic slides less rich in pollen. Because pollen grains are 3-D shapes, catching all important features can only be achieved using different focal levels, so-called ‘Z-stacks’. A total of 20 Z-stacks were used in this study with a step size of 1.8 µm. The settings used for scanning were a Plan Apochromat 100× (oil) objective and numerical aperture 0.55 with a brightfield contrast manager. To maintain similar conditions in the image collection process, the condenser was always set to 3.3 V with an exposure time of 28 ms.

### Reference pollen image library

All images were post-processed in ImageJ v1.52a (Fiji)^46^ using the script Pollen_Projector (https://github.com/pollingmarcel/Pollen_Projector). The input for this script is a folder containing all raw pollen images (including all Z-stacks), and the output is a set of projections for each individual pollen grain that are subsequently used as input for the deep learning model.

Pollen_Projector identifies all complete, non-overlapping pollen grains and extracts them as stacks from the raw Z-stack. This is achieved using binarization on the raw images to detect only those rounded objects with a circularity > 0.3 and a size larger than 5 µm*.* Out-of-focus images within each group of 20 Z-stack slices were removed using a threshold for minimum and maximum pixel values. The conventional input of a convolutional neural network is a three-channel image. In colour images RGB channels are commonly used, but since we use grayscale images, three different Z-stack projections were chosen to represent the three different channels. The projections used are Standard Deviation, Minimum Intensity and Extended Focus. Standard Deviation creates an image containing the standard deviation of the pixel intensities through the stack, where positions with large differences appear brighter in the final projection. Minimum intensity takes the minimum pixel value through the stack and uses that for the projection. Finally, the Extended Focus projection was created using the ‘Extended_Depth_of_Field’ ImageJ macro of Richard Wheeler (www.richardwheeler.net)^47^. This macro takes a stack of images with a range of focal depths and builds a 2D image from it using only in focus regions of the images. A schematic overview of the processes behind the Pollen_Projector script is shown in Supplementary Fig. S2. Finally, to keep the original size information of the pollen grains they were inserted into a 276 × 276 frame.

### Convolutional neural networks

Convolutional Neural Networks (CNNs) are widely used in the field of computer vision for image classification, object detection, facial recognition, autonomous driving, etc. For this study we used the VGG16 network^48^, MobileNetV1^49^ and MobileNetV2^50^ in Keras^51^. Compared with traditional neural networks and shallow convolutional neural networks, VGG16 has deeper layers that extract more representative features from images (Fig. 2a). In contrast, MobileNets are small low-power models that offer a time-efficient alternative. A feature extractor and classifier are two key structural parts of the CNN that perform the classification task. The VGG16 network contains 13 convolutional layers that form five blocks, which generate features from images in the feature extraction phase. Subsequently, three fully connected (FC) layers were built and added to the convolutional layers to classify the different classes (Supplementary Fig. S3). The MobileNetV1 uses depth-wise separable convolutions to build light weight deep neural networks. It has 28 layers in total. A final average pooling reduces the spatial resolution to 1 and connected with FC and Softmax layer for classification^49^. MobileNetV2, which has 53 layers, is an improved version of MobileNetV1 by introducing inverted residual structure and linear bottleneck layers^50^. MobileNetV2 is more accurate than MobileNetV1 and can be much faster. We trained classification models based on aforementioned CNNs using our pollen image dataset.

During the training process, the initial parameters of convolutional layers were derived from the pre-trained network on the ImageNet dataset. Subsequently, the convolutional layers and the following fully connected layers were further fine-turned based on our own image dataset so as to classify the different classes. The pre-trained models were compared to models trained from scratch. In order to avoid overfitting, we compared the results of five- and tenfold cross-validation in the training process. For fivefold cross-validation the pollen image dataset is split into a training and validation data set in the ratio 80/20 while this is 90/10 for tenfold cross-validation. For each fold, the number of epochs was set to 30. The accuracy of the model converged at this point and the model is therefore found not to be overfitting (Supplementary Fig. S4).

In order to quantify model accuracy, several commonly used performance measures were used:

$$precision=\frac{TP}{TP+FP}$$

$$recall=\frac{TP}{TP+FN}$$

$$F1 score=2*\frac{precision*recall}{precision+recall}$$

$$CCR=\frac{TP+TN}{TP+TN+FP+FN}$$

where *TP* refers to True Positives, *TN* to True Negatives, *FP* to False Positives and *FN* to False Negatives. Recall is the number of True Positives divided by the total number of elements that belong to the correct class, which is the sum of the True Positives and False Negatives. The F1-score is the weighted average of the precision and recall. The correct classification rate (CCR) reflects the accuracy of the model. The values represent the average weighted by the number of images in each class.

### Data augmentation

A large number of images for each class is required to train a deep learning model, as the performance will increase when more variation is fed to the model. Due to the nature of the images investigated in this study, the model was sensitive to small changes, since the differences between the pollen grains are very subtle. Therefore, data augmentation was used to increase the variety of pollen images used as input. We selected the augmentation options brightness and flip. These options were used since size and shape of pollen are key features for their identification, and using other augmentation options would artificially change the original morphology of the pollen grains. Brightness range was set from 0.1 to 2, with < 1 corresponding to a darker image and > 1 to a brighter image. Horizontal- and vertical flip were also applied randomly (Supplementary Fig. S5). In addition, we applied L2 regularization and dropout in our neural network structures to prevent overfitting.

### Test cases

For each aerobiological sample an area representing 10% of the total deposition area was scanned manually for Urticaceae pollen grains (i.e. eight full transects at 100× magnification) resulting in 112 pollen grains from the sample from Leiden (LUMC, the Netherlands), 63 from Lleida and 26 from Vielha (both ICTA-UAB, Catalonia, Spain). One aspect of the Catalonian aerobiological samples was the presence of pollen from families that produce pollen similar to Urticaceae, that are rarely encountered in the Netherlands. These included *Humulus lupulus* L. (Cannabaceae) and *Morus* sp. (Moraceae) which were not included in our training dataset. These can be distinguished from Urticaceae, however, in the case of *H. lupulus* by their much larger size (up to 35 µm) and the very large onci and, in the case of *Morus* by the more ellipsoidal shape. These pollen grains were removed from the dataset before they were fed to the CNN for classification.

## Supplementary Information

Supplementary Information.

## Data Availability

All data generated or analyzed during this study are included in this published article (and its “Supplementary Information” files). Raw pollen images can be made available upon request.
