# Supplementary material for: Neural networks for increased accuracy of allergenic pollen monitoring
Source: Sci Rep. 2021 May 31;11:11357. doi: 10.1038/s41598-021-90433-x (PMC8166864; doi:10.1038/s41598-021-90433-x)
Supplement: Supplementary file 1 — Supplementary Information. [file 41598_2021_90433_MOESM1_ESM.docx]

**Supplementary Information**

**Neural networks for increased accuracy of allergenic pollen monitoring**

Marcel Polling^*^ and Chen Li, Lu Cao, Fons Verbeek, Letty A. de Weger, Jordina Belmonte, Concepción De Linares, Joost Willemse, Hugo de Boer, Barbara Gravendeel

* Corresponding author, [marcel.polling@naturalis.nl](mailto:marcel.polling@naturalis.nl)

# Supplementary Table S1

Probability scores for Urticaceae pollen grains scanned from aerobiological samples using the pre-trained VGG16 model with 5-fold cross-validation. *U. mem* = *Urtica membranacea*

## Lleida (16-06-2019), n = 63

| **Image No.** | **Probability *Parietaria*** | **Probability *Urtica*** | **Probability**  ***U. mem*** | **Final ID (threshold 0.6)** | **Final ID (threshold 0.7)** |
| --- | --- | --- | --- | --- | --- |
| 1 | 0.95 | 0.05 | 0.00 | *Parietaria* | *Parietaria* |
| 2 | 0.98 | 0.02 | 0.00 | *Parietaria* | *Parietaria* |
| 3 | 0.29 | 0.70 | 0.01 | *Urtica* | *Urtica* |
| 4 | 0.98 | 0.02 | 0.00 | *Parietaria* | *Parietaria* |
| 5 | 0.24 | 0.76 | 0.00 | *Urtica* | *Urtica* |
| 6 | 1.00 | 0.00 | 0.00 | *Parietaria* | *Parietaria* |
| 7 | 0.94 | 0.06 | 0.00 | *Parietaria* | *Parietaria* |
| 8 | 0.99 | 0.01 | 0.00 | *Parietaria* | *Parietaria* |
| 9 | 0.12 | 0.88 | 0.00 | *Urtica* | *Urtica* |
| 10 | 0.99 | 0.01 | 0.00 | *Parietaria* | *Parietaria* |
| 11 | 0.99 | 0.01 | 0.00 | *Parietaria* | *Parietaria* |
| 12 | 0.96 | 0.04 | 0.00 | *Parietaria* | *Parietaria* |
| 13 | 1.00 | 0.00 | 0.00 | *Parietaria* | *Parietaria* |
| 14 | 0.96 | 0.04 | 0.00 | *Parietaria* | *Parietaria* |
| 15 | 1.00 | 0.00 | 0.00 | *Parietaria* | *Parietaria* |
| 16 | 0.90 | 0.09 | 0.01 | *Parietaria* | *Parietaria* |
| 17 | 0.90 | 0.01 | 0.09 | *Parietaria* | *Parietaria* |
| 18 | 0.73 | 0.16 | 0.10 | *Parietaria* | *Parietaria* |
| 19 | 0.95 | 0.04 | 0.00 | *Parietaria* | *Parietaria* |
| 20 | 0.98 | 0.00 | 0.02 | *Parietaria* | *Parietaria* |
| 21 | 0.16 | 0.83 | 0.00 | *Urtica* | *Urtica* |
| 22 | 0.67 | 0.31 | 0.02 | *Parietaria* | unknown |
| 23 | 0.02 | 0.98 | 0.00 | *Urtica* | *Urtica* |
| 24 | 0.95 | 0.04 | 0.00 | *Parietaria* | *Parietaria* |
| 25 | 0.99 | 0.01 | 0.00 | *Parietaria* | *Parietaria* |
| 26 | 0.99 | 0.01 | 0.00 | *Parietaria* | *Parietaria* |
| 27 | 0.95 | 0.05 | 0.00 | *Parietaria* | *Parietaria* |
| 28 | 0.02 | 0.98 | 0.00 | *Urtica* | *Urtica* |
| 29 | 0.34 | 0.66 | 0.00 | *Urtica* | unknown |
| 30 | 1.00 | 0.00 | 0.00 | *Parietaria* | *Parietaria* |
| 31 | 0.98 | 0.02 | 0.00 | *Parietaria* | *Parietaria* |
| 32 | 1.00 | 0.00 | 0.00 | *Parietaria* | *Parietaria* |
| 33 | 0.99 | 0.01 | 0.00 | *Parietaria* | *Parietaria* |
| 34 | 0.92 | 0.02 | 0.06 | *Parietaria* | *Parietaria* |
| 35 | 0.57 | 0.41 | 0.02 | unknown | unknown |
| 36 | 1.00 | 0.00 | 0.00 | *Parietaria* | *Parietaria* |
| 37 | 0.87 | 0.13 | 0.00 | *Parietaria* | *Parietaria* |
| 38 | 0.99 | 0.00 | 0.00 | *Parietaria* | *Parietaria* |
| 39 | 0.97 | 0.03 | 0.00 | *Parietaria* | *Parietaria* |
| 40 | 0.58 | 0.41 | 0.01 | unknown | unknown |
| 41 | 0.98 | 0.02 | 0.00 | *Parietaria* | *Parietaria* |
| 42 | 0.70 | 0.29 | 0.00 | *Parietaria* | *Parietaria* |
| 43 | 0.84 | 0.16 | 0.00 | *Parietaria* | *Parietaria* |
| 44 | 0.97 | 0.02 | 0.01 | *Parietaria* | *Parietaria* |
| 45 | 0.83 | 0.17 | 0.00 | *Parietaria* | *Parietaria* |
| 46 | 0.99 | 0.00 | 0.00 | *Parietaria* | *Parietaria* |
| 47 | 1.00 | 0.00 | 0.00 | *Parietaria* | *Parietaria* |
| 48 | 0.99 | 0.00 | 0.00 | *Parietaria* | *Parietaria* |
| 49 | 0.96 | 0.04 | 0.01 | *Parietaria* | *Parietaria* |
| 50 | 0.00 | 1.00 | 0.00 | *Urtica* | *Urtica* |
| 51 | 0.99 | 0.01 | 0.00 | *Parietaria* | *Parietaria* |
| 52 | 0.99 | 0.00 | 0.01 | *Parietaria* | *Parietaria* |
| 53 | 0.91 | 0.04 | 0.05 | *Parietaria* | *Parietaria* |
| 54 | 0.95 | 0.04 | 0.00 | *Parietaria* | *Parietaria* |
| 55 | 0.90 | 0.10 | 0.00 | *Parietaria* | *Parietaria* |
| 56 | 0.95 | 0.05 | 0.00 | *Parietaria* | *Parietaria* |
| 57 | 0.99 | 0.01 | 0.00 | *Parietaria* | *Parietaria* |
| 58 | 1.00 | 0.00 | 0.00 | *Parietaria* | *Parietaria* |
| 59 | 0.99 | 0.01 | 0.00 | *Parietaria* | *Parietaria* |
| 60 | 0.17 | 0.82 | 0.00 | *Urtica* | *Urtica* |
| 61 | 0.41 | 0.56 | 0.02 | unknown | unknown |
| 62 | 0.98 | 0.02 | 0.00 | *Parietaria* | *Parietaria* |
| 63 | 0.76 | 0.21 | 0.03 | *Parietaria* | *Parietaria* |

## Vielha, 09-08-2019, n = 26

| Image No. | Probability *Parietaria* | Probability *Urtica* | Probability  *U. mem* | Final ID (threshold 0.6) | Final ID (threshold 0.7) |
| --- | --- | --- | --- | --- | --- |
| 1 | 0.03 | 0.97 | 0.00 | *Urtica* | *Urtica* |
| 2 | 0.07 | 0.86 | 0.07 | *Urtica* | *Urtica* |
| 3 | 0.10 | 0.90 | 0.00 | *Urtica* | *Urtica* |
| 4 | 0.02 | 0.98 | 0.00 | *Urtica* | *Urtica* |
| 5 | 0.09 | 0.91 | 0.00 | *Urtica* | *Urtica* |
| 6 | 0.26 | 0.74 | 0.00 | *Urtica* | *Urtica* |
| 7 | 0.00 | 1.00 | 0.00 | *Urtica* | *Urtica* |
| 8 | 0.41 | 0.04 | 0.55 | unknown | unknown |
| 9 | 0.61 | 0.39 | 0.01 | *Parietaria* | unknown |
| 10 | 0.81 | 0.10 | 0.09 | *Parietaria* | *Parietaria* |
| 11 | 0.02 | 0.98 | 0.00 | *Urtica* | *Urtica* |
| 12 | 0.01 | 0.99 | 0.00 | *Urtica* | *Urtica* |
| 13 | 0.49 | 0.13 | 0.38 | unknown | unknown |
| 14 | 0.00 | 1.00 | 0.00 | *Urtica* | *Urtica* |
| 15 | 0.14 | 0.84 | 0.02 | *Urtica* | *Urtica* |
| 16 | 0.63 | 0.10 | 0.27 | *Parietaria* | unknown |
| 17 | 0.12 | 0.88 | 0.00 | *Urtica* | *Urtica* |
| 18 | 0.09 | 0.90 | 0.00 | *Urtica* | *Urtica* |
| 19 | 0.24 | 0.76 | 0.00 | *Urtica* | *Urtica* |
| 20 | 0.04 | 0.96 | 0.00 | *Urtica* | *Urtica* |
| 21 | 0.85 | 0.12 | 0.03 | *Parietaria* | *Parietaria* |
| 22 | 0.80 | 0.14 | 0.07 | *Parietaria* | *Parietaria* |
| 23 | 0.00 | 1.00 | 0.00 | *Urtica* | *Urtica* |
| 24 | 0.17 | 0.83 | 0.00 | *Urtica* | *Urtica* |
| 25 | 0.02 | 0.98 | 0.00 | *Urtica* | *Urtica* |
| 26 | 0.57 | 0.43 | 0.00 | unknown | unknown |

## Leiden (23-08-2019), n = 112

| **Image No.** | **Probability *Parietaria*** | **Probability *Urtica*** | **Probability**  ***U. mem*** | **Final ID (threshold 0.6)** | **Final ID (threshold 0.7)** |
| --- | --- | --- | --- | --- | --- |
| 1 | 0.04 | 0.96 | 0.00 | *Urtica* | *Urtica* |
| 2 | 0.01 | 0.99 | 0.00 | *Urtica* | *Urtica* |
| 3 | 0.07 | 0.93 | 0.00 | *Urtica* | *Urtica* |
| 4 | 0.16 | 0.83 | 0.00 | *Urtica* | *Urtica* |
| 5 | 0.19 | 0.81 | 0.00 | *Urtica* | *Urtica* |
| 6 | 0.02 | 0.98 | 0.00 | *Urtica* | *Urtica* |
| 7 | 0.00 | 1.00 | 0.00 | *Urtica* | *Urtica* |
| 8 | 0.28 | 0.72 | 0.00 | *Urtica* | *Urtica* |
| 9 | 0.11 | 0.89 | 0.00 | *Urtica* | *Urtica* |
| 10 | 0.34 | 0.66 | 0.00 | *Urtica* | unknown |
| 11 | 0.04 | 0.96 | 0.00 | *Urtica* | *Urtica* |
| 12 | 0.18 | 0.81 | 0.00 | *Urtica* | *Urtica* |
| 13 | 0.00 | 1.00 | 0.00 | *Urtica* | *Urtica* |
| 14 | 0.47 | 0.53 | 0.00 | unknown | unknown |
| 15 | 0.11 | 0.89 | 0.00 | *Urtica* | *Urtica* |
| 16 | 0.01 | 0.99 | 0.00 | *Urtica* | *Urtica* |
| 17 | 0.20 | 0.80 | 0.00 | *Urtica* | *Urtica* |
| 18 | 0.00 | 1.00 | 0.00 | *Urtica* | *Urtica* |
| 19 | 0.00 | 1.00 | 0.00 | *Urtica* | *Urtica* |
| 20 | 0.01 | 0.99 | 0.00 | *Urtica* | *Urtica* |
| 21 | 0.75 | 0.25 | 0.00 | *Parietaria* | *Parietaria* |
| 22 | 0.00 | 1.00 | 0.00 | *Urtica* | *Urtica* |
| 23 | 0.03 | 0.97 | 0.00 | *Urtica* | *Urtica* |
| 24 | 0.01 | 0.99 | 0.00 | *Urtica* | *Urtica* |
| 25 | 0.69 | 0.31 | 0.00 | *Parietaria* | unknown |
| 26 | 0.11 | 0.89 | 0.00 | *Urtica* | *Urtica* |
| 27 | 0.12 | 0.88 | 0.00 | *Urtica* | *Urtica* |
| 28 | 0.17 | 0.83 | 0.00 | *Urtica* | *Urtica* |
| 29 | 0.09 | 0.91 | 0.00 | *Urtica* | *Urtica* |
| 30 | 0.00 | 1.00 | 0.00 | *Urtica* | *Urtica* |
| 31 | 0.48 | 0.52 | 0.00 | unknown | unknown |
| 32 | 0.24 | 0.76 | 0.00 | *Urtica* | *Urtica* |
| 33 | 0.06 | 0.94 | 0.00 | *Urtica* | *Urtica* |
| 34 | 0.29 | 0.71 | 0.00 | *Urtica* | *Urtica* |
| 35 | 0.14 | 0.86 | 0.00 | *Urtica* | *Urtica* |
| 36 | 0.38 | 0.62 | 0.00 | *Urtica* | unknown |
| 37 | 0.06 | 0.94 | 0.00 | *Urtica* | *Urtica* |
| 38 | 0.55 | 0.45 | 0.00 | unknown | unknown |
| 39 | 0.01 | 0.99 | 0.00 | *Urtica* | *Urtica* |
| 40 | 0.00 | 1.00 | 0.00 | *Urtica* | *Urtica* |
| 41 | 0.00 | 1.00 | 0.00 | *Urtica* | *Urtica* |
| 42 | 0.02 | 0.98 | 0.00 | *Urtica* | *Urtica* |
| 43 | 0.03 | 0.97 | 0.00 | *Urtica* | *Urtica* |
| 44 | 0.21 | 0.79 | 0.00 | *Urtica* | *Urtica* |
| 45 | 0.02 | 0.98 | 0.00 | *Urtica* | *Urtica* |
| 46 | 0.00 | 1.00 | 0.00 | *Urtica* | *Urtica* |
| 47 | 0.01 | 0.99 | 0.00 | *Urtica* | *Urtica* |
| 48 | 0.79 | 0.20 | 0.00 | *Parietaria* | *Parietaria* |
| 49 | 0.54 | 0.46 | 0.00 | unknown | unknown |
| 50 | 0.01 | 0.99 | 0.00 | *Urtica* | *Urtica* |
| 51 | 0.00 | 1.00 | 0.00 | *Urtica* | *Urtica* |
| 52 | 0.01 | 0.99 | 0.00 | *Urtica* | *Urtica* |
| 53 | 0.01 | 0.99 | 0.00 | *Urtica* | *Urtica* |
| 54 | 0.00 | 1.00 | 0.00 | *Urtica* | *Urtica* |
| 55 | 0.00 | 1.00 | 0.00 | *Urtica* | *Urtica* |
| 56 | 0.00 | 1.00 | 0.00 | *Urtica* | *Urtica* |
| 57 | 0.02 | 0.98 | 0.00 | *Urtica* | *Urtica* |
| 58 | 0.00 | 1.00 | 0.00 | *Urtica* | *Urtica* |
| 59 | 0.54 | 0.46 | 0.00 | unknown | unknown |
| 60 | 0.45 | 0.55 | 0.00 | unknown | unknown |
| 61 | 0.09 | 0.91 | 0.00 | *Urtica* | *Urtica* |
| 62 | 0.00 | 1.00 | 0.00 | *Urtica* | *Urtica* |
| 63 | 0.00 | 1.00 | 0.00 | *Urtica* | *Urtica* |
| 64 | 0.00 | 1.00 | 0.00 | *Urtica* | *Urtica* |
| 65 | 0.06 | 0.94 | 0.00 | *Urtica* | *Urtica* |
| 66 | 0.05 | 0.95 | 0.00 | *Urtica* | *Urtica* |
| 67 | 0.01 | 0.99 | 0.00 | *Urtica* | *Urtica* |
| 68 | 0.23 | 0.77 | 0.00 | *Urtica* | *Urtica* |
| 69 | 0.21 | 0.79 | 0.00 | *Urtica* | *Urtica* |
| 70 | 0.72 | 0.28 | 0.00 | *Parietaria* | *Parietaria* |
| 71 | 0.49 | 0.51 | 0.00 | unknown | unknown |
| 72 | 0.06 | 0.94 | 0.00 | *Urtica* | *Urtica* |
| 73 | 0.33 | 0.67 | 0.00 | *Urtica* | unknown |
| 74 | 0.00 | 1.00 | 0.00 | *Urtica* | *Urtica* |
| 75 | 0.28 | 0.72 | 0.00 | *Urtica* | *Urtica* |
| 76 | 0.00 | 1.00 | 0.00 | *Urtica* | *Urtica* |
| 77 | 0.03 | 0.97 | 0.00 | *Urtica* | *Urtica* |
| 78 | 0.05 | 0.95 | 0.00 | *Urtica* | *Urtica* |
| 79 | 0.21 | 0.79 | 0.00 | *Urtica* | *Urtica* |
| 80 | 0.00 | 1.00 | 0.00 | *Urtica* | *Urtica* |
| 81 | 0.00 | 1.00 | 0.00 | *Urtica* | *Urtica* |
| 82 | 0.03 | 0.97 | 0.00 | *Urtica* | *Urtica* |
| 83 | 0.02 | 0.98 | 0.00 | *Urtica* | *Urtica* |
| 84 | 0.12 | 0.88 | 0.00 | *Urtica* | *Urtica* |
| 85 | 0.17 | 0.83 | 0.00 | *Urtica* | *Urtica* |
| 86 | 0.01 | 0.99 | 0.00 | *Urtica* | *Urtica* |
| 87 | 0.90 | 0.10 | 0.00 | *Parietaria* | *Parietaria* |
| 88 | 0.11 | 0.89 | 0.00 | *Urtica* | *Urtica* |
| 89 | 0.02 | 0.98 | 0.00 | *Urtica* | *Urtica* |
| 90 | 0.00 | 1.00 | 0.00 | *Urtica* | *Urtica* |
| 91 | 0.29 | 0.71 | 0.00 | *Urtica* | *Urtica* |
| 92 | 0.11 | 0.89 | 0.00 | *Urtica* | *Urtica* |
| 93 | 0.12 | 0.88 | 0.00 | *Urtica* | *Urtica* |
| 94 | 0.01 | 0.99 | 0.00 | *Urtica* | *Urtica* |
| 95 | 0.01 | 0.99 | 0.00 | *Urtica* | *Urtica* |
| 96 | 0.00 | 1.00 | 0.00 | *Urtica* | *Urtica* |
| 97 | 0.00 | 1.00 | 0.00 | *Urtica* | *Urtica* |
| 98 | 0.02 | 0.98 | 0.00 | *Urtica* | *Urtica* |
| 99 | 0.00 | 1.00 | 0.00 | *Urtica* | *Urtica* |
| 100 | 0.21 | 0.79 | 0.00 | *Urtica* | *Urtica* |
| 101 | 0.55 | 0.45 | 0.00 | unknown | unknown |
| 102 | 0.00 | 1.00 | 0.00 | *Urtica* | *Urtica* |
| 103 | 0.48 | 0.52 | 0.00 | unknown | unknown |
| 104 | 0.00 | 1.00 | 0.00 | *Urtica* | *Urtica* |
| 105 | 0.00 | 1.00 | 0.00 | *Urtica* | *Urtica* |
| 106 | 0.57 | 0.43 | 0.00 | unknown | unknown |
| 107 | 0.11 | 0.89 | 0.00 | *Urtica* | *Urtica* |
| 108 | 0.23 | 0.77 | 0.00 | *Urtica* | *Urtica* |
| 109 | 0.26 | 0.74 | 0.00 | *Urtica* | *Urtica* |
| 110 | 0.12 | 0.88 | 0.00 | *Urtica* | *Urtica* |
| 111 | 0.58 | 0.42 | 0.00 | unknown | unknown |
| 112 | 0.00 | 1.00 | 0.00 | *Urtica* | *Urtica* |

# Supplementary Figure S1


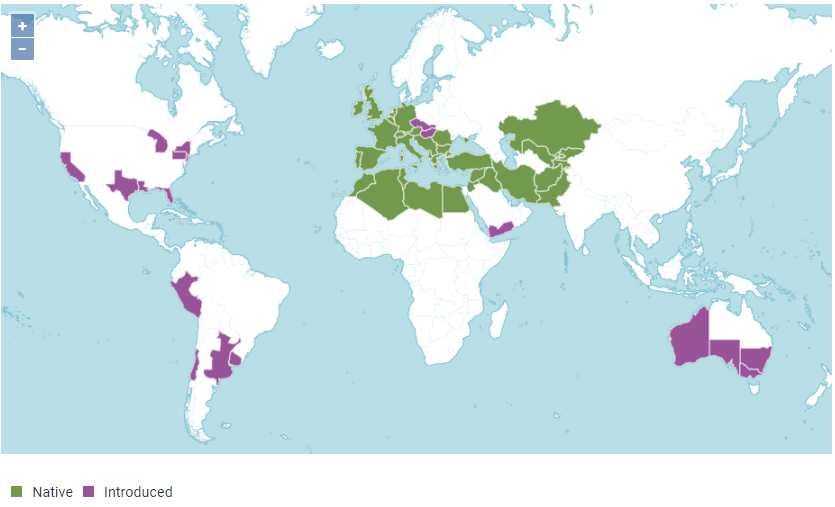

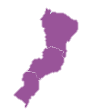

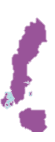

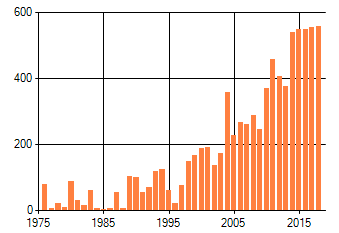


**(a)**

**(b)**

**Figure S1**. **(a)** Global native (green) and introduced (purple) distribution of *Parietaria judaica* and *P. officinalis* (POWO (2019). "Plants of the World Online. Map taken from the Royal Botanic Gardens, Kew. http://www.plantsoftheworldonline.org/ Retrieved 05 October 2020"). **(b)** Trend in Pellitory of the wall (*Parietaria judaica*) plant sightings per square kilometre in the Netherlands over the past 45 years. Index number = 100 for 1990 © NEM (CBS & FLORON) 2019.

# Supplementary Table S2

Locations of all Urticaceae specimens and number of images. NL = the Netherlands, SP = Spain and PO = Portugal. *collected in 2018 and 2019, deposited in the Naturalis Biodiversity Center herbarium.

| **Species**  **(n = total images)** | **Geographical origin** | **Collection date** | **No. of images used** | **Deposition number** |
| --- | --- | --- | --- | --- |
| *Parietaria judaica* L.  (n = 1670) | Montejaque (SP)  Leiden, Stationsweg (NL)  Huizen (NL)  Leiden, Robijnstraat (NL)  Den Haag (NL)  Leiden, Paterstraatje  Sassenplaat (NL)  Rotterdam, Hartelkanaal (NL) | 17/10/2011  19/11/2019  20/09/2014  23/07/2012  05/10/2018  09/10/2018  03/07/2013  27/09/2014 | 54  168  174  139  392  250  233  260 | WAG.1186948  L.3993376*  L.4303913  L.2071680  L.3993377*  L.3993378*  L.4304093  L.4304136 |
| *Parietaria officinalis* L.  (n = 1359) | Middelburg (NL)  Haarlem (NL)  Wageningse Polder (NL)  Leiden (NL)  Den Haag, Escamplaan (NL)  Den Haag, Bosjes van Poot (NL) | 26/06/2014  13/07/2013  19/07/2012  07/2012  12/10/2018  01/08/2012 | 234  191  64  369  383  248 | L.3974371  L.2073373  WAG.1186992  L.3963901  L.3993379*  L.2071818 |
| *Urtica dioica* L.  (n = 1055) | Leiden, Hogeschool 1 (NL)  Leiden, Hogeschool 2 (NL)  Den Haag (NL)  Leiden, Sandifortdreef (NL)  Arnhem (NL) | 06/11/2019  07/11/2019  17/11/2019  15/11/2019  29/05/2001 | 316  299  182  191  67 | L.3993380*  L.3993381*  L.3993382*  L.3993383*  WAG.1188104 |
| *Urtica membranacea* Poir. ex Savigny  (n = 1118) | Amsterdam (NL)  Overloon (NL)  Cape st. Vincent (PO)  Den Haag (NL) | 11/2018  17/06/2014  03/1995  06/03/2019 | 521  135  87  375 | L.3993384*  L.3959964  L.1629741  L.3993385* |
| *Urtica urens* L.  (n = 1270) | Leiden (NL)  Castilla-la-Mancha (SP)  Zandvoort (NL)  Meijendel (NL)  Zwolle (NL)  Wassenaar (NL) Den Haag (NL) | 01/11/2019  27/05/2016  05/08/2012  12/08/2011  29/04/2005  15/09/2002 13/03/2020 | 128  165  201  140  134  219 283 | L.3993386*  WAG.1962413  L.2071917  L.2074446  L.4271105  L.4233917 L.3993387* |

# Supplementary Figure S2


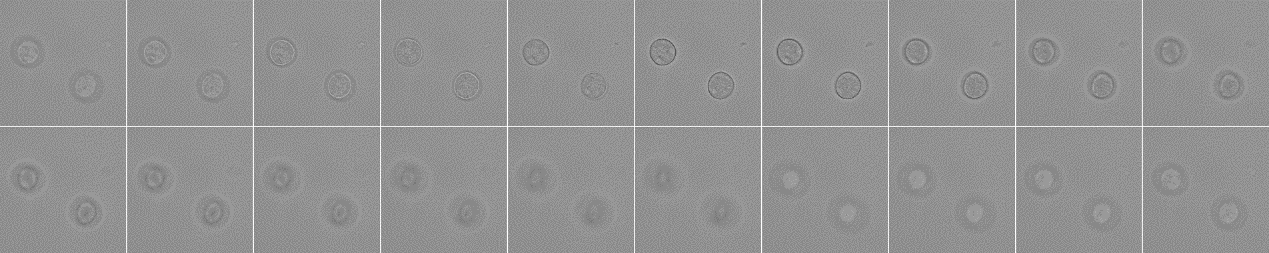


Z=1

Z=10

Z=20

Z=11


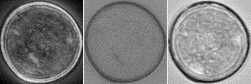

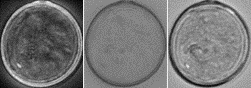

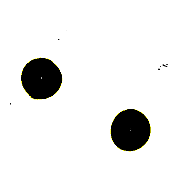

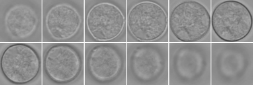

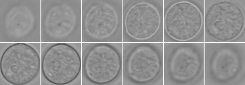


Cropping out pollen in a stack, using only in-focus informative images

Output: Three Z-stack projections for each pollen grain (STD, MIN, EXT)

Input: Raw images (20 Z-slices) containing pollen

STD

MIN

EXT

STD

MIN

EXT

**Figure S2.** Pollen image acquisition and processing workflow carried out with in-house designed Pollen_Projector script. Once raw images are obtained at 20 different focal levels (‘Z-slices’), subsequent steps involve cropping of whole individual pollen grains and producing three different projections from the Z-stacks. Abbreviations of projections: STD = Standard Deviation, MIN = Minimum Intensity and EXT = Extended Focus.

# Supplementary Figure S3

**Figure S3.** Schematic overview of the structure of VGG-16 with an example of three-channel input image of a *Parietaria judaica* pollen grain (known label) and the output generated, where it confidently identifies the images as *Parietaria* (98% probability). Adapted from Simonyan et al., (2014)^1^


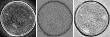

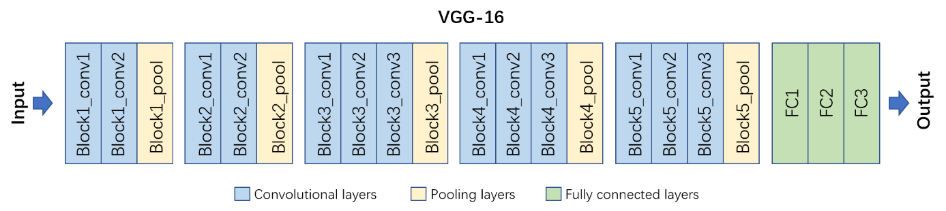


*Parietaria* (98.5%)

*Urtica*
(1.5%)

­­­­­­­

# Supplementary Figure S4


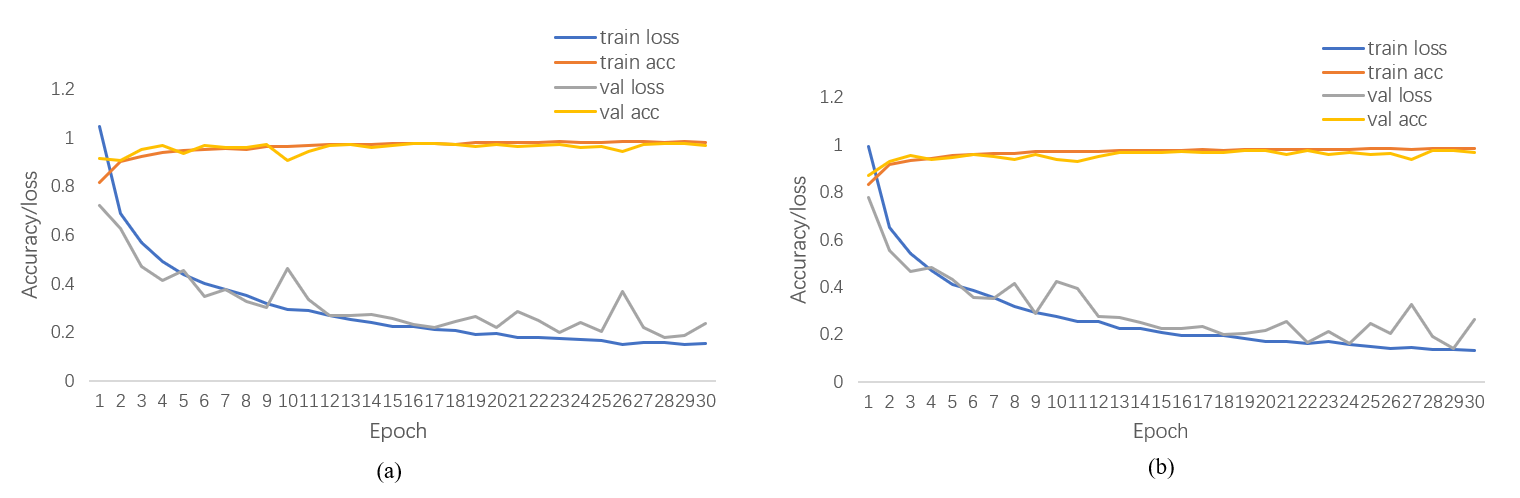


**Figure S4.** Figures showing the accuracy/loss plots for the VGG16 model with 5- and 10-fold cross-validation.

# Supplementary Figure S5


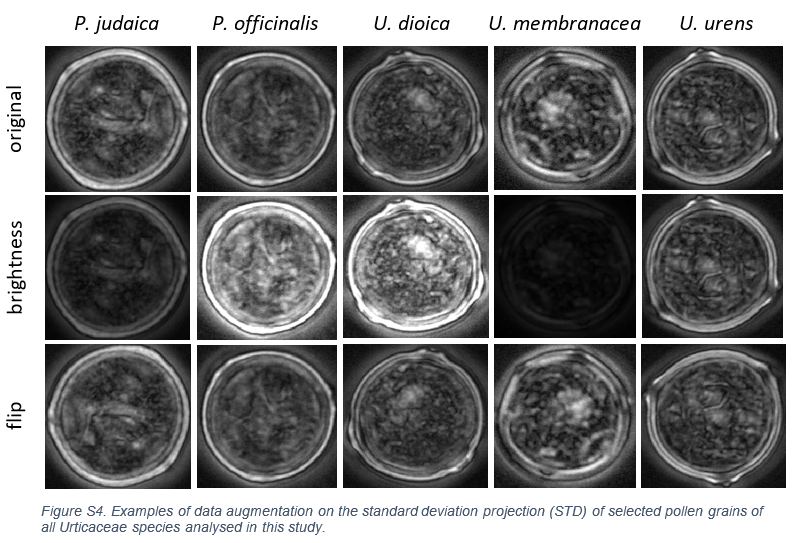


**Figure S5.** Examples of data augmentation on the Standard Deviation Projection (STD) of selected pollen grains of all Urticaceae pollen species used in this study.

# Supplementary reference

1 Simonyan, K. & Zisserman, A. Very deep convolutional networks for large-scale image recognition. *arXiv preprint arXiv:1409.1556* (2014).
